# Combined supplementation of citric acid and oregano essential oil enhanced growth performance, digestion, immunity, and resistance against *Nocardia seriolae* in spotted sea bass (*Lateolabrax maculatus*) fed high soybean meal diet

**DOI:** 10.3389/fphys.2026.1921084

**Published:** 2026-09-11

**Authors:** Haohang Fang, Hanle Zhang, Jianxue Wang, Lei Wang, Biao Yun, Xueqiao Qian, Shouqi Xie

**Affiliations:** 1 Key Laboratory of Microecological Resources and Utilization in Breeding Industry, Ministry of Agriculture and Rural Affairs, Guangdong Haid Group Co., Ltd., Guangzhou, Guangdong, China; 2 Key Laboratory of Breeding Biotechnology and Sustainable Aquaculture, Institute of Hydrobiology, Chinese Academy of Sciences, Wuhan, China; 3 Zhuhai Rongchuan Feed Co., Ltd., Zhuhai, Guangdong, China

**Keywords:** citric acid, growth performance, *Lateolabrax maculatus*, Nocardia seriolae, oregano essential oil

## Abstract

This study evaluated the individual and combined effects of citric acid and oregano essential oil on growth performance, digestive capacity, immune responses, and disease resistance against *Nocardia seriolae* in spotted sea bass (*Lateolabrax maculatus*) fed a high soybean meal (SBM) diet. Five isonitrogenous and isolipidic diets were formulated: a positive control diet (LS, 0% soybean meal), a negative control diet (HS, 30% soybean meal), and three supplemented diets based on HS, including 1% citric acid (HSC), 0.3% oregano essential oil (HSO), and their combination (HSCO). Fish (initial weight around 68 ag) were fed for 8 weeks, followed by a 20-day *N. seriolae* challenge trial. Results showed that compared with the LS group, fish fed with the HS diet significantly reduced weight gain and lipase activity, while upregulating the mRNA expression of pro-inflammatory cytokines in the mid-intestine. On the contrary, supplementation with oregano essential oil, alone or in combination with citric acid, significantly improved growth performance, whereas citric acid alone exerted no significant improvement on growth. Nevertheless, all three additive groups effectively enhanced lipase activity and suppressed the transcription of intestinal pro-inflammatory genes compared to the HS group. Notably, the HSCO group exhibited significantly higher C3 and C4 levels than both HSC and HSO groups, indicating a superior combined effect on complement activation, and achieved a survival rate (55%) after *N. seriolae* challenge relative to the HS group (20%), which was close to that of the LS group (65%). Overall, dietary supplementation with 1% citric acid and 0.3% oregano essential oil effectively mitigated the adverse impacts in *L. maculatus* fed with high soybean meal diets.

## Introduction

Fish meal is regarded as the optimal protein source in aquafeeds because of the balanced nutritional profile. However, rising fishmeal prices and supply shortages have spurred an ongoing search for sustainable alternative protein sources (Olsen and Hasan, 2012). Among plant-based proteins, soybean meal (SBM) is widely used as a substitute due to its high protein content, stable supply, and affordable price (Zhou et al., 2018). Nevertheless, high dietary inclusion of SBM introduces anti-nutritional factors such as phytate and trypsin inhibitors, which can suppress growth performance and reduce nutritional utilization, thereby restricting its application in carnivorous fish species such as spotted sea bass (*Lateolabrax maculatus*) (Zhou et al., 2018).

*L. maculatus* is favored by the Chinese customers due to its rapid growth and delicious flesh (Huang et al., 2025). However, intensive aquaculture often accompanies disease outbreaks, resulting in significant economic losses (Behringer et al., 2020). Among them, nocardiosis (induced by *Nocardia seriolae*) is a chronic granulomatous disease with high morbidity, posing a serious threat to the *L. maculatus* aquaculture industry (Dong et al., 2024). Up to now, there has been little research on nutritional strategies that can improve dietary utilization and enhance bacterial resistance in this species fed with high soybean meal diet.

Citric acid, an organic acidifier, has been reported to reduce gastrointestinal pH, promote phytate hydrolysis, and improve nutrient utilization in fish (Khajepour and Hosseini, 2012). Its primary mode of action is enhancing digestibility and absorption of dietary nutrients. Previous studies in *Colossoma macropomum* (Nascimento et al., 2021) and *Pagrus major* (Sarker et al., 2007) have demonstrated that dietary citric acid supplementation improves growth performance, enhances digestive enzyme activities, and alleviates the detrimental effects induced by plant protein based diets. In contrast, oregano essential oil is a natural plant extract with primarily immunomodulatory and antimicrobial properties in various fish studies (Alagawany et al., 2020). Its bioactive components, carvacrol and thymol, have been shown to regulate inflammatory signaling and exert antibacterial effects. Reports in *Oreochromis niloticus* (Shourbela et al., 2021) and *Cyprinus carpio L.* (Abdel-Latif et al., 2020) showed that oregano essential oil promotes growth, enhances immune parameters, and increases resistance against pathogenic bacterial infections.

Due to the improvement of nutrient utilization by citric acid and enhancement of immunoregulatory effects by oregano essential oil, their combined application may produce synergistic effects in fish. However, to our knowledge, no prior study has systematically evaluated the single and combined effects of citric acid and oregano essential oil on growth, digestive function, intestinal immunity, and resistance against *N. seriolae* infection in *L. maculatus* fed high-SBM diets. The present study was conducted to evaluate the individual and combined effects of dietary supplementation of citric acid and oregano essential oil on growth performance, digestive function, intestinal immunity, and resistance against *N. seriolae* infection in *L. maculatus*, aiming to provide a theoretical basis for *L. maculatus* culture.

## Materials and methods

### Experimental diets

Five isonitrogenous and isolipidic experimental diets were formulated and analyzed (**Table 1**). A diet without soybean meal was designed as the positive control group (LS). In comparison, the HS diet was formulated by replacing 24% of fishmeal (reduced from 48% in LS to 24% in HS) with 30% soybean meal on an isonitrogenous and isolipidic basis. Based on the HS diet, three additive treatments were supplemented with 1% citric acid (HSC), 0.3% oregano essential oil (HSO), and a combination of 1% citric acid and 0.3% oregano essential oil (HSCO), respectively. Microcrystalline cellulose was included at varying levels across diets to compensate for differences in ingredient inclusion. The supplemental levels of citric acid (1%) (Hossain et al., 2007; Pandey and Satoh, 2008) and oregano essential oil (0.3%) (Ahmadifar et al., 2011; Diler et al., 2017) were refereed to other carnivorous fish. Citric acid (purity ≥ 99.5%) and oregano essential oil were purchased from Shanghai Aladdin Biochemical Technology Co., Ltd. (China). Dietary ingredients were obtained from Guangdong Haid Group Co., Ltd. (China).

**Table 1:** Ingredient and analysis compositions of the experimental diets.

	LS	HS	HSC	HSO	HSCO
Ingredients (%)					
Fish meal	48	24	24	24	24
Soybean meal	0	30	30	30	30
Poultry by product meal	20	26	26	26	26
Wheat flour	15	4.5	4.5	4.5	4.5
Fish oil	5	5.7	5.7	5.7	5.7
Soybean oil	2	2	2	2	2
Soybean phospholipid	2	2	2	2	2
Vitamin premix	2	2	2	2	2
Mineral premix	2	2	2	2	2
DL-Methionine	0	0.17	0.17	0.17	0.17
L-Lysine	0	0.25	0.25	0.25	0.25
Microcrystalline cellulose	4	1.38	0.38	1.08	0.08
Citric Acid	0	0	1	0	1
Oregano oils	0	0	0	0.3	0.3
Sum	100	100	100	100	100
Analysis Compositions (%)					
Crude protein	48.27	48.18	48.16	48.22	48.15
Crude lipid	14.89	15.04	15.08	14.98	14.86
Moisture	7.24	7.12	7.07	7.28	7.16

Citric acid (Purity≥99.5%) and oregano oils were purchased from Shanghai Aladdin Biochemical Technology Co., Ltd. (China), and the other ingredients were obtained from Guangdong Haid Group Co., Ltd. (China).

All ingredients were mixed using a stepwise dilution method. For the oregano essential oil-containing diets (HSO and HSCO), oregano essential oil was first thoroughly mixed with the soybean oil portion to ensure uniform dispersion before being added to the ingredient mixture. Afterward, fish oil, soybean oil, soybean lecithin, and deionized water were added, and the mixture was blended for an additional 10 min. The blended mash was processed into 3-mm extruded pellets using a multifunctional spiral extruder (CD4XITS, South China University of Technology, Guangzhou, China). The pellets were dried in an oven at 60 °C until moisture content below 8%, and then stored at −20 °C.

### Fish feeding and management

The feeding trial was conducted at the Aquaculture Research and Development Headquarters of Haid Group (Guangzhou, China). *L. maculatus* with an initial average body weight of approximately 68 ag were randomly distributed into five dietary groups, with four replicate tanks per group and 30 fish per tank (1 m³ volume). All tanks were supplied with an indoor recirculating aquaculture system.

Prior to the initiation of the experiment, all fish were acclimated to the experimental conditions for two weeks and fed a commercial diet (Haid Group). During the 8-week feeding trial, fish were hand-fed to apparent satiation twice daily at 08:00 and 16:30. Water temperature was maintained at 27 ± 1 °C, dissolved oxygen at 6 ± 0.5 mg/L, and ammonia nitrogen below 0.2 mg/L throughout the trial. A natural photoperiod was applied.

### Sampling

After 8-week feeding trial, fish were fasted for 24 ah. During sampling, fish were anesthetized with MS-222 (1:10,000; Sigma-Aldrich, USA). Total number and total weight of fish in each tank were recorded. Three fish were randomly selected from each tank, and their samples were pooled into a single composite sample per tank.

Blood samples were collected from the caudal vein using 1 mL sterile syringes and transferred into 1.5 mL sterile centrifuge tubes. Blood was allowed to clot at room temperature for 2 ah, then centrifuged at 3000 × g for 10 min at 4 °C to obtain serum. After blood collection, mid-intestinal samples were collected: one portion was frozen in liquid nitrogen, while another portion was fixed in 4% paraformaldehyde.

### *Nocardia seriolae* challenge test

After feeding trial, five fish from each replicate tank were picked and combined into one tank (total 20 fish per treatment) for the bacterial challenge test. The *N. seriolae* strain was isolated and identified by Haid Research Institute from diseased cultured fish. Prior to the challenging test, a preliminary experiment was conducted to determine the median lethal dose (LD_50_: 4.8 × 10^4^ CFU per fish) of *N. seriolae* for *L. maculatus* (**Supplementary Figure 1**). All fish were intraperitoneally injected with 0.1 mL of bacterial suspension containing 4.8 × 10^5^ CFU/ml (0.1 mL × 4.8 × 10^5^ CFU/mL = 4.8 × 10^4^ CFU per fish), which equals the pre-determined LD_50_ (1 × LD_50_). The LD_50_ dose was chosen to enable clear differentiation of resistance levels among treatment groups, as lower doses may produce insufficient mortality to detect differences, while higher doses may overwhelm host defenses and mask treatment effects. Dead fish were recorded within 20 days of the challenge test and promptly removed to prevent water contamination. During the challenging period, water temperature was ranged: 27.0–29.2 °C (**Supplementary Figure 2**). This range is within the optimal thermal window for *N. seriolae* infection in *L. maculatus*, thus not confounding the survival results.

### Chemical analysis

Dietary proximate composition was analyzed according to standard methods (Thiex, 2019). Moisture was determined by drying samples to constant weight at 105 °C. Crude protein was measured using the Kjeldahl method (N × 6.25) after acid digestion. Crude lipid was determined by petroleum ether extraction after acid hydrolysis.

Serum complement C3 (H186-1), complement C4 (H186-2), and lysozyme (A050-1) activities, as well as mid-intestinal lipase (A054-2-1) and amylase (C016-2-1) activities, were measured using commercial assay kits (Nanjing Jiancheng Bioengineering Institute, China). For digestive enzyme analysis, mid-intestinal samples were homogenized in physiological saline and centrifuged (4 °C), and the supernatant was collected for analysis.

### Quantitative real time PCR analysis

Total RNA was extracted from mid-intestinal tissues using TRIzol^®^ reagent (Invitrogen, USA) following the manufacturer’s protocol. RNA concentration and purity (A260/280 ratios: around 2.0) were tested using a NanoDrop 2000 spectrophotometer. cDNA was synthesized using a PrimeScript™ RT reagent Kit (Takara, Japan).

Quantitative real-time PCR (qRT-PCR) was performed using SYBR^®^ Premix Ex Taq™ II (Takara, Japan) on a LightCycler 480 system (Roche Applied Science, Switzerland). Each 20 μL reaction mixture contained 10 μL SYBR Premix Ex Taq II, 0.4 μL of each forward and reverse primer, 2 μL cDNA template, and 7.2 μL sterile water. The amplification program was set as followed: 95 °C for 30 s; 95 °C for 5 s and 60 °C for 30 s (40 cycles). All samples were analyzed in duplicate.

The *β-actin* gene was used as the house keeping gene, and the expression level of targeted gene were calculated using the 2^−ΔΔCt^ method. Primer sequences were referred to previous study (Zhang et al., 2018) and listed in **Table 2**.

**Table 2:** Primers of candidate genes for qPCR analysis.

Target genes	Forward primer (5′-3′)	Reverse primer (5′-3′)	Annealing temperature (°C)
House-keeping genes			
*β-Actin*	AACTGGGATGACATGGAGAAG	TTGGCTTTGGGGTTCAGG	60
Nutrient transporter genes			
*SLC1A5*	GACGTGCGGTCCACAAAATG	TCATCCTGGCTGTTGACTGG	60
*LAT1*	TGGCCTACTTCACCACCATT	CGGAGTGAAGAGGTCTGTGT	60
*PEPT1*	GACTTCTGCAGCTGACTTCG	TCGGCCCAAAGTCAAAAGTG	60
Pro-inflammatory genes			
*TNF-α*	GATCGTCATCCCACAAACCG	GCTTTGCTGCCTATGGAGTC	60
*IL-1β*	GTCAACTTACGTGCACCCTG	AAATCGTACCATGTCGCTGC	60
*IL-2*	GGGAAAGTGTCACATGTCCG	ACGGCCTGGTTTAAAGATGC	60
*IL-8*	GGATCAGTTTCTTCACCCAGG	CAGGTGGAGTCGAGGATCAT	60
Anti-inflammatory cytokine genes			
*IL-4*	ACCATGCATTACTACAGCACTG	CACATTCAGGGGCGTTTGTC	60

*SLC1A5*, Solute Carrier Family 1 Member 5; *LAT1*, L-type amino acid transporter 1; *PEPT1*, Peptide Transporter 1; *TNF-α*, Tumor Necrosis Factor-α; *IL-1β*, Interleukin-1 β; *IL-2*, Interleukin-2; *IL-8*, Interleukin-8; *IL-4*, Interleukin-4.

### Intestinal morphology

Mid-intestinal samples were fixed in 4% paraformaldehyde for 24 ah, dehydrated in a graded ethanol series, cleared in xylene, and embedded in paraffin. Serial sections of 5 μm thickness were cut using a microtome, mounted on glass slides, and stained with hematoxylin and eosin (H&E) (Krogdahl et al., 2003). After staining, sections were sealed with neutral balsam. Images were captured under a light microscope, and villus height and muscularis thickness were measured. For each intestinal cross-section, 8 intact villi with clearly visible lamina propria and muscular layers were randomly selected and measured. One cross-section per tank was analyzed (n = 4 tanks per treatment), and the measurements from all 8 villi within the same section were averaged to obtain a single replicate value.

### Statistical analysis

Data are presented as mean ± standard error (SE). Prior to statistical analysis, normality of data distribution was verified using the Shapiro–Wilk test, and homogeneity of variances was confirmed using Levene’s test. Statistical analysis for continuous variables (growth performance, digestive enzyme activities, serum immune parameters, gene expression, and intestinal morphology) was performed using one-way analysis of variance (ANOVA) followed by Duncan’s multiple range test. Survival data from the *N. seriolae* challenge test were analyzed using the Log-rank (Mantel–Cox) test to compare survival curves among treatment groups. Differences were considered significant at p < 0.05.

## Results

### Growth performance

Growth performance of *L. maculatus* fed with experimental diets for 56 days is presented in **Table 3**. Compared to the LS group, fish fed the HS diet showed significantly lower weight gain (WG) (p < 0.05). In contrast, dietary supplementation with oregano essential oil, either alone (HSO) or in combination with citric acid (HSCO), significantly improved WG than the HS group (p < 0.05). No significant difference in WG was observed between the HSC and HS groups (p > 0.05). Meanwhile, survival rate (SR) and feed conversion ratio (FCR) showed no significant differences among all treatments (p > 0.05).

**Table 3:** Growth performance of *L. maculatus* fed with different diets for 56 days.

	LS	HS	HSC	HSO	HSCO
IW	68.17±0.54	68.83±0.82	68.7±0.91	68.37±0.89	68.48±0.79
FW	200.06±6.28a	174.65±6.02c	186.55±6.16bc	187.8±3.98ab	188.08±6.16ab
WG	193.49±8.82a	153.77±8.43c	171.49±6.09bc	174.75±7.57b	174.67±9.64b
SR	97.5±3.19	99.17±1.67	98.33±1.92	99.17±1.67	100±0
FCR	1.05±0.01	1.07±0.03	1.04±0.02	1.05±0.03	1.04±0.01

IBW (g per fish): initial body weight.

FBW (g per fish): final body weight.

Weight gain (WG, %) = 100 × (final body weight – initial body weight)/initial body weight.

Survival rate (SR) (%) = 100 × (final number of fish)/ (initial number of fish).

Feed conversion ratio (FCR) = diet fed/ weight gain.

Values are presented as mean ± SE (n=4). Values in the same row having different superscript letters are significantly different (*P* < 0.05).

### Mid-intestinal digestive enzyme activities

As shown in **Table 4**, mid-intestinal lipase activity of fish fed with HS diet was significantly lower than that in the LS group (p < 0.05). In comparison, fish fed with HSC, HSO, or HSCO diets significantly improved lipase activity compared to the HS group (p < 0.05). However, no significant differences in amylase activity were observed among all experimental groups (p > 0.05).

**Table 4:** Midgut digestive enzymes of *L. maculatus* fed with different diets for 56 days.

	LS	HS	HSC	HSO	HSCO
Amylase (U/mg prot)	0.78±0.12	0.51±0.25	0.55±0.18	0.63±0.26	0.69±0.14
Lipase (U/mg prot)	257.7±18.5a	160.3±24.7b	198.3±23.6c	220.1±10.2c	214.6±14.7c

Values are presented as mean ± SE (n=4). Values in the same row having different superscript letters are significantly different (*P* < 0.05).

### Mid-intestinal nutrient transporter gene expression

**Figure 1:**
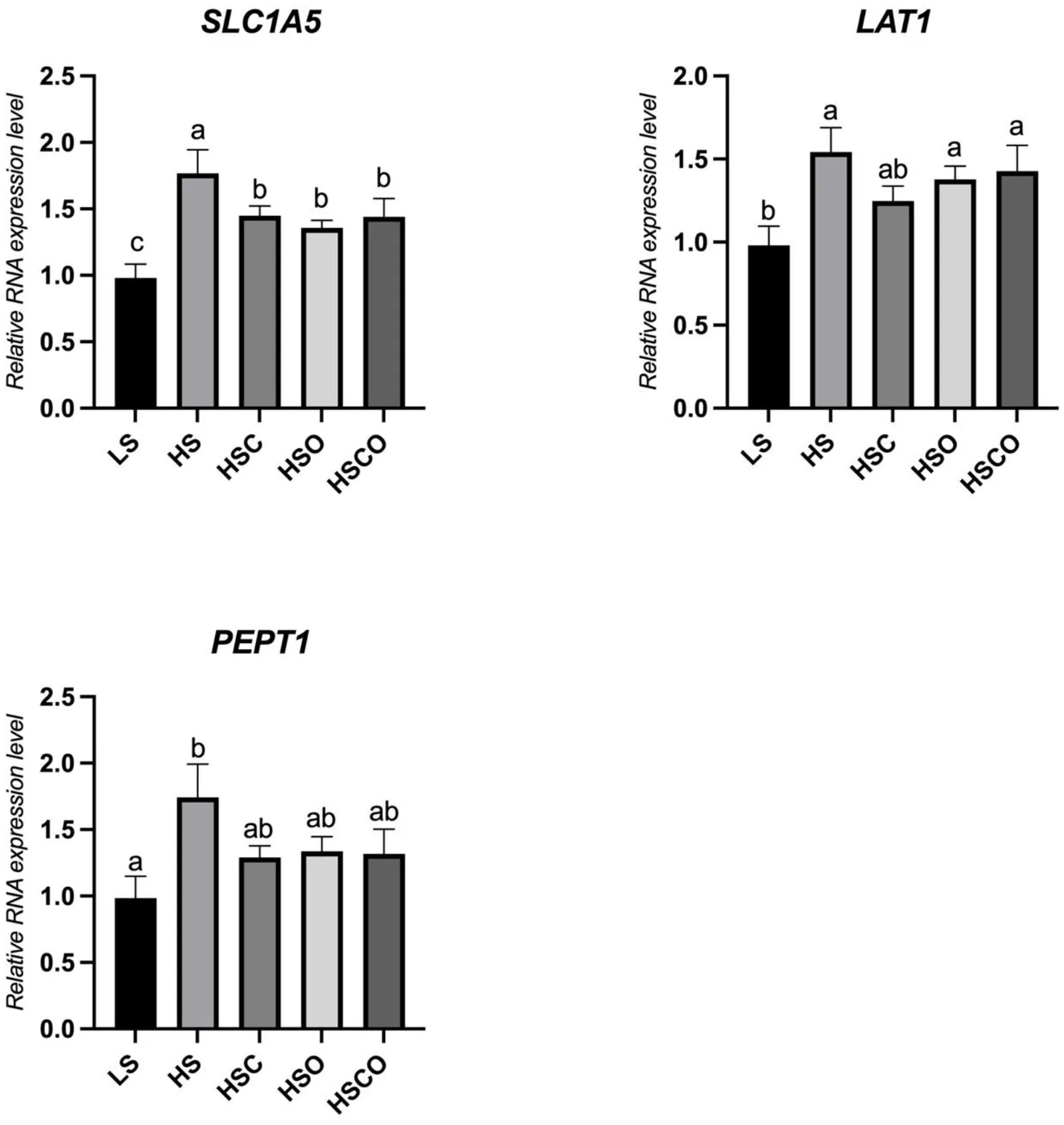
Mid-intestine mRNA expression levels of nutrient transporter related genes of *L. maculatus* fed with different diets for 56 days. The small letters indicated significant differences at *p < 0.05*. Values are mean ± SE (n = 4).

Fish fed the HS diet showed significantly higher mid-intestinal mRNA expression levels of *SLC1A5*, *LAT1*, and *PEPT1* than those in the LS group (p < 0.05) (**Figure 1**). For *SLC1A5*, all three supplemented groups (HSC, HSO, and HSCO) showed significantly higher values than the HS group (p < 0.05). No significant differences in *LAT1* and *PEPT1* mRNA levels were observed between the supplemented groups and the HS group (p > 0.05).

### Mid-intestinal histomorphology

**Figure 2:**
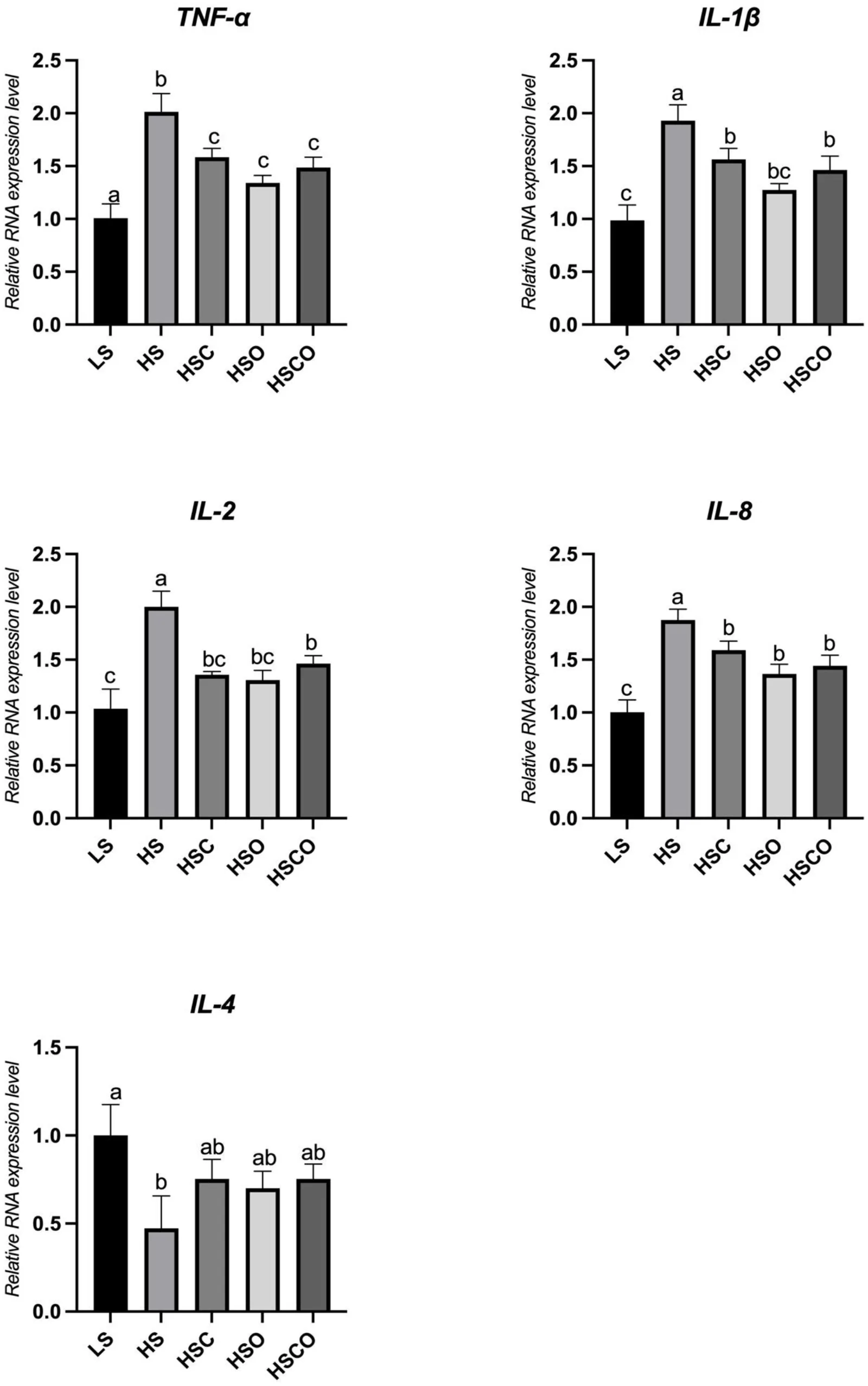
Light microscopy of mid-intestine morphology of *L. maculatus* fed with different diets (Pictures (LS): LS group; Pictures (HS): HS group; Pictures (HSC): HSC group; Pictures (HSO): HSO group; Pictures (HSCO): HSCO group) for 56 days. Scale bars of pictures are 500 μm.

As shown in **Figure 2** and **Table 5**, no significant differences in intestinal villus height were observed among all groups (p > 0.05). However, fish in the HSO group showed significantly greater muscularis thickness compared with the HS group (p < 0.05).

**Table 5:** Mid-intestine morphology of *L. maculatus* fed with different diets for 56 days.

	LS	HS	HSC	HSO	HSCO
Villus height (μm)	821.7±75.1	744.3±62.2	887.3±111.9	794±62.6	774.3±92.5
Muscularis thickness (μm)	203.3±28.4ab	151.7±32.5a	185±20.2ab	229±27.6b	178.7±13.7ab

Values are presented as mean ± SE (n=4). Values in the same row having different superscript letters are significantly different (*P* < 0.05).

### Intestinal immune-related gene expression

**Figure 3:**
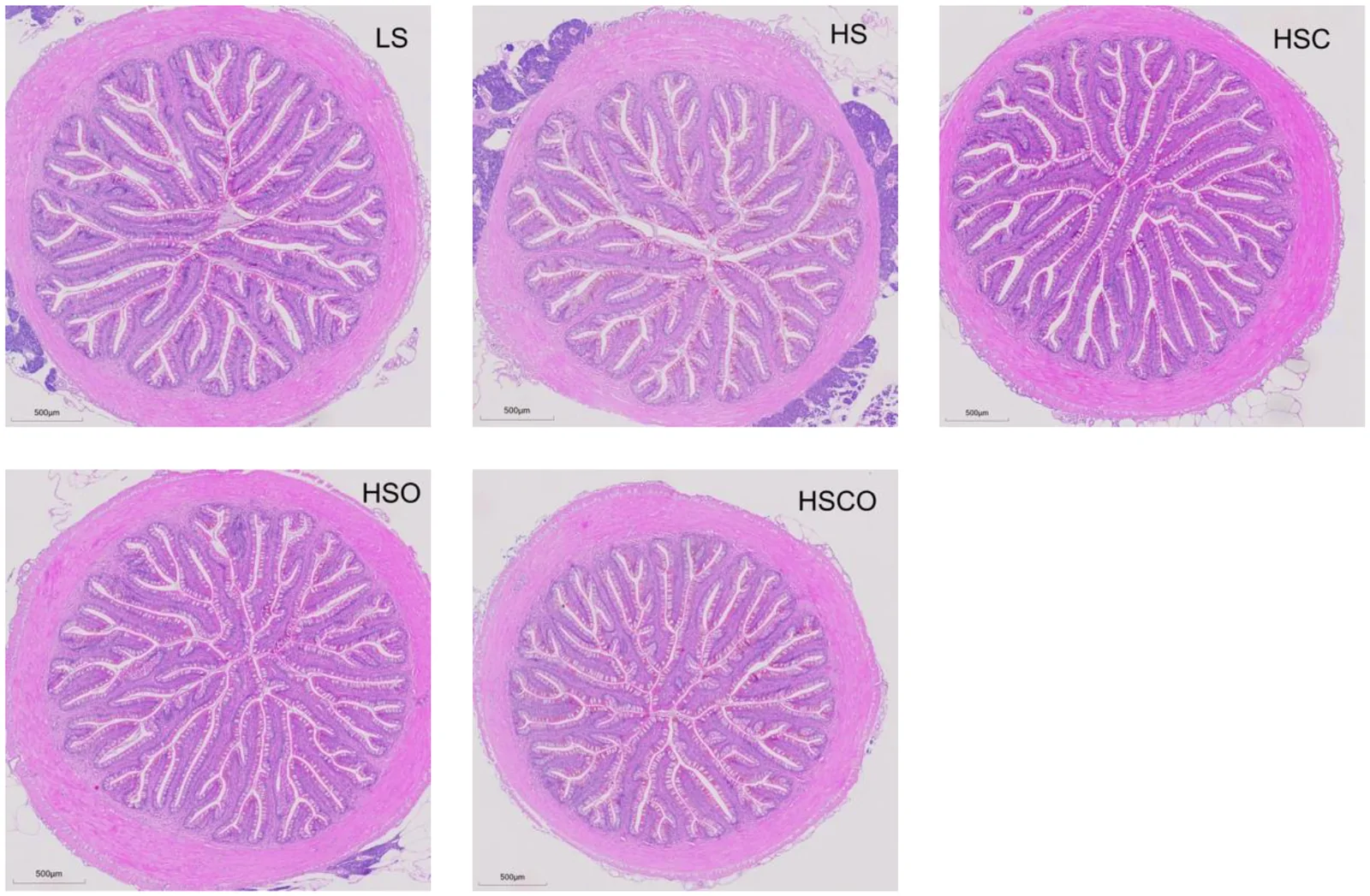
Mid-intestine mRNA expression levels of pro-inflammatory and anti-inflammatory genes of *L. maculatus* fed with different diets for 56 days. The small letters indicated significant differences at *p < 0.05*. Values are mean ± SE (n = 4).

The mRNA expression levels of pro-inflammatory cytokines (*TNF-α*, *IL-1β*, *IL-2*, and *IL-8*) in the mid-intestine were significantly upregulated in the HS group compared to the LS group (p < 0.05) (**Figure 3**). In contrast, fish fed with HSC, HSO, or HSCO diets significantly downregulated the transcription of these four genes compared with the HS group (p < 0.05).

For the anti-inflammatory cytokine, *IL-4* mRNA expression was significantly reduced in the HS group than in the LS group (p < 0.05). No significant difference in *IL-4* expression was observed between any additive groups (HSC, HSO, HSCO) and the HS group (p > 0.05).

### Non-specific immune parameters

No significant differences in serum complement C3, C4, or lysozyme activity were observed between the LS and HS groups (p > 0.05) (**Table 6**). In addition, the HSC diet significantly increased serum C3, C4, and lysozyme levels compared to the HS group (p < 0.05). The HSO diet significantly improved serum C3 and lysozyme activities (p < 0.05) but did not significantly affect C4 (p > 0.05) compared to the HS group. Furthermore, the HSCO diet not only significantly increased C3, C4, and lysozyme levels compared with the HS group (p < 0.05), but also further enhanced C3 and C4 contents compared to the HSC and HSO groups individually (p < 0.05), suggesting a superior combined effect on the complement system.

**Table 6:** Plasma nonspecific immune system parameters of *L. maculatus* fed with different diets for 56 days.

	LS	HS	HSC	HSO	HSCO
Complement C3 (ug/mL)	2±0.1bc	1.8±0.1c	2.4±0.1b	2.2±0.1b	3.9±0.2a
Complement C4 (ug/mL)	8.4±0.4c	8.2±0.1c	10.2±0.3b	8.9±0.1c	13±0.6a
Lysozyme (U/mL)	221.7±22.7ab	167±7.8b	227.7±30.2a	231.6±19a	246.3±21.9a

Values are presented as mean ± SE (n=4). Values in the same row having different superscript letters are significantly different (*P* < 0.05).

### Survival rate following nocardia seriolae challenge

**Figure 4:**
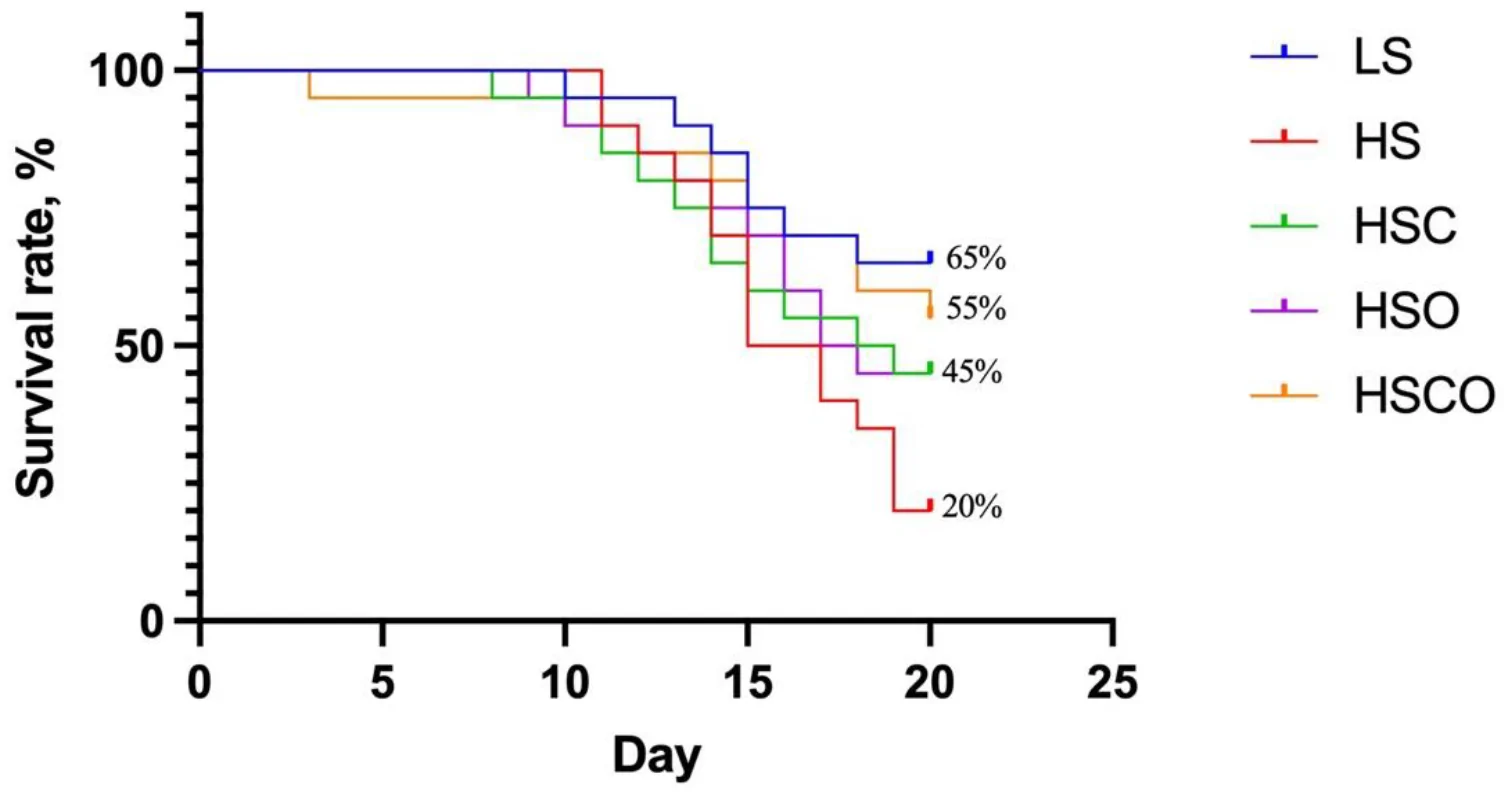
Survival curve of *L. maculatus* fed different diets within 20 days post *N. seriolae* challenge.

After bacterial challenge, the LS group showed a survival rate of 65%, whereas the HS group exhibited the lowest survival rate at only 20% (**Figure 4**). Supplementation with citric acid (HSC) or oregano essential oil (HSO) alone improved survival rates to 45% in both groups. Notably, fish in the HSCO group achieved a survival rate of 55%, which was higher than that of the HS group and close to the LS group.

## Discussion

In the present study, fish fed the 30% soybean meal diet exhibited significantly lower weight gain compared with those fed the diet without soybean meal. This is consistent with the findings of Liu et al (Liu et al., 2024a, b), who reported that increasing dietary soybean meal from 16% to 40% suppressed growth in *L. maculatus.* Notably, supplementation with oregano essential oil alone or in combination with citric acid significantly improved weight gain of *L. maculatus*, whereas citric acid alone showed no significant improvement. This result indicates that oregano essential oil played a key role in promoting fish growth, which is consistent with results in zebrafish (Dissinger et al., 2024), where supplementation with 2% or 3% oregano essential oil in high soybean meal diets significantly improved growth performance. On the contrary, citric acid alone (HSC) did not improve weight gain. This may be attributed to the 30% soybean meal inclusion level exceeding the threshold at which citric acid alone can effectively counteract the anti-nutritional effects. In contrast, combined supplementation with oregano oil provided both digestive enhancement and anti-inflammatory benefits, which collectively contributed to growth recovery.

In the present study, lipase activity was significantly lower in the HS group than in the LS group, indicating that increasing dietary plant protein suppress pancreatic digestive enzyme secretion (Zhang et al., 2018). Further, our results showed that supplementation with oregano essential oil significantly restored lipase activity inhibited by the high soybean meal diet. Similarly, a study in common carp found that dietary oregano essential oil supplementation significantly increased intestinal digestive enzyme activity (Zhang et al., 2020). In addition, the present study demonstrated that oregano essential oil supplementation significantly increased mid-intestinal muscularis thickness. Increased muscularis thickness enhances intestinal motility and digestive capacity (Gao et al., 2013). Similarly, a positive linear effect of oregano oil on intestinal muscularis thickness was also observed in *Astyanax altiparanae* (Ferreira et al., 2016). Oregano essential oil, particularly its bioactive components carvacrol and thymol, has been associated with increased digestive enzyme activity and improved intestinal morphology in fish (Kolygas et al., 2025). In contrast to lipase, amylase activity was not affected by dietary treatments. This is consistent with the carnivorous feeding habit, which relies primarily on lipids and proteins rather than carbohydrates as energy sources. Carnivorous species have relatively low amylase secretion capacity, and the soybean meal inclusion in the present study may not have imposed sufficient carbohydrate load to affect amylase expression.

Interestingly, mRNA expression levels of nutrient transporter genes (*SLC1A5*, *LAT1*, and *PEPT1*) were significantly upregulated in the HS group compared with the LS group, which represents a compensatory response to reduced nutrient digestibility (Brezas et al., 2021). Similar compensatory upregulation has been observed in *Lateolabrax japonicus* fed high plant protein diets, where replacing fish meal (21% of feed) with soybean meal (30% of feed) significantly upregulated mid-intestinal *SLC1A5* and *LAT1* expression (Zhang et al., 2018). This conserved response across *Lateolabrax* species may represent a general adaptive strategy to plant-protein-induced nutrient stress. Compared with the HS group, all three additive-supplemented groups showed further upregulation of *SLC1A5* expression, suggesting enhanced amino acid transport capacity (Zhang et al., 2025). However, *LAT1* and *PEPT1* expression did not affect among the additive-supplemented groups, suggesting gene-specific regulatory mechanisms.

In the present study, fish fed the HS diet exhibited significantly higher mRNA expression levels of *TNF-α*, *IL-1β*, *IL-2*, and *IL-8* in the mid-intestine compared with those fed the LS diet, while the anti-inflammatory cytokine IL-4 was significantly downregulated. These findings are consistent with previous studies in different fish species, demonstrating that high soybean meal diets induce intestinal inflammation (Liu et al., 2024a; Oladipupo et al., 2024; Su et al., 2025). In contrast, all three additive-supplemented groups (HSC, HSO, and HSCO) significantly downregulated mRNA expression of these pro-inflammatory cytokines. Similarly, dietary citric acid supplementation significantly reduced the expression of pro-inflammatory genes such as *TNF-α* and *IFN-γ* in *Scophthalmus maximus L.* fed high soybean meal diets (Zhao et al., 2019). In *Cyprinus carpio L.*, dietary oregano essential oil supplementation significantly regulated immune-related gene expression in the liver, including upregulation of *IL-1β* and *IL-10* (Abdel-Latif et al., 2020). Studies in *Oreochromis niloticus* also demonstrated that oregano essential oil supplementation modulated the expression of immune-related genes such as *TNF-α* and *IL-1β*, thereby improving intestinal health (Moustafa et al., 2025). However, IL-4 mRNA expression, which was significantly reduced in the HS group, did not recover in any of the additive groups. This suggests that the anti-inflammatory effects of citric acid and oregano oil in the mid-intestine are primarily mediated through suppression of pro-inflammatory cytokines rather than through upregulation of IL-4.

The challenge test results showed that the HS group exhibited a survival rate of only 20% at 20 days post-challenge with *N. seriolae*, which was lower than that of the LS group (65%). The reduced disease resistance in the HS group may be associated with intestinal inflammation. Chronic intestinal inflammation can impair intestinal barrier function, increase the risk of pathogen invasion, and consequently reduce host resistance to systemic infections (Han et al., 2023; Wu et al., 2024; Wang et al., 2025). Intestinal inflammation may compromise barrier function and increase pathogen translocation risk, although this hypothesis requires direct testing. Dietary supplementation with citric acid or oregano essential oil, either individually or in combination, effectively enhanced resistance of *L. maculatus* against *N. seriolae*, with the combined supplementation group (HSCO) achieving a survival rate of 55%, approaching the level observed in the LS group. The improved survival may be attributed to enhanced complement and lysozyme systems. Complement C3 and C4 plays critical roles in innate immunity by eliminating foreign microorganisms through opsonization, inflammatory chemotaxis, and direct pathogen killing (Zarkadis et al., 2001). Lysozyme exerts antibacterial effects by hydrolyzing peptidoglycan in bacterial cell walls (Jing et al., 2025). Similarly, compared to control treatment, rainbow trout (*Oncorhynchus mykiss*) fed diets supplemented with oregano oil exhibited significantly elevated plasma lysozyme activity, and reduced mortality rates attacked by *Lactococcus garvieae* (Diler et al., 2017). Also, studies in common carp confirmed that supplementation with oregano essential oil (3.0 mL/kg) significantly increased survival rate following *Aeromonas hydrophila* challenge (Yigit et al., 2024).

A noteworthy finding is the superior elevation of serum complement C3 and C4 levels in the HSCO group compared to either HSC or HSO alone. A possible mechanistic explanation is as follows. Citric acid improves dietary nutrient digestibility and absorption, thereby providing an enhanced supply of amino acid precursors for hepatic complement protein synthesis. Concurrently, oregano oil alleviates intestinal inflammation, reducing the systemic inflammatory burden that may otherwise suppress hepatic acute-phase protein production. The convergence of enhanced substrate availability and attenuated immune inhibition may jointly contribute to the amplified serum complement production. However, this hypothesis requires further investigation.

The limitations of this study should be acknowledged. First, only single fixed dosages of citric acid (1%) and oregano essential oil (0.3%) were tested in this study, selected based on previous studies in other carnivorous fish species. As the optimal dosages for *L. maculatus* may differ, graded dose–response trials are warranted to establish potential dose-dependent effects. Second, the 8-week feeding trial was followed by a single sampling time point, which precludes assessment of the temporal dynamics of digestive response. Multi-timepoint sampling in future studies would facilitate a more comprehensive understanding of the adaptive process to high-SBM diets. Third, intestinal microbiota composition was not characterized in this study. Metagenomic analysis would help determine whether the observed effects are mediated, at least in part, through microbiota modulation.

## Conclusion

Overall, dietary 0.3% oregano essential oil supplementation effectively alleviated the negative effects of high soybean meal diets on growth performance, digestive capacity, and intestinal health in *Lateolabrax maculatus*. Notably, combined supplementation with 1% citric acid and 0.3% oregano essential oil showed superior effects in improving the survival rate of fish following *Nocardia seriolae* challenge compared to either additive alone.

## Data Availability

The raw data supporting the conclusions of this article will be made available by the authors, without undue reservation.
