# Supplementary material for: Combined supplementation of citric acid and oregano essential oil enhanced growth performance, digestion, immunity, and resistance against Nocardia seriolae in spotted sea bass (Lateolabrax maculatus) fed high soybean meal diet
Source: Front Physiol. 2026 Sep 11;17:1921084. doi: 10.3389/fphys.2026.1921084 (PMC13612341; doi:10.3389/fphys.2026.1921084)
Supplement: Supplementary file 1 [file SupplementaryFile1.docx]

Cumulative mortality of Lateolabrax maculatus after Nocardia seriolae challenge

|  | PBS | 5*10^6 | 5*10^5 | 5*10^4 | 5*10^3 | 5*10^2 |
| --- | --- | --- | --- | --- | --- | --- |
| 1d | 0 | 0 | 0 | 0 | 0 | 0 |
| 2d | 0 | 0 | 0 | 0 | 0 | 0 |
| 3d | 0 | 0 | 0 | 0 | 0 | 0 |
| 4d | 0 | 3 | 0 | 0 | 0 | 0 |
| 5d | 0 | 3 | 0 | 0 | 0 | 0 |
| 6d | 0 | 3 | 0 | 0 | 0 | 0 |
| 7d | 0 | 3 | 0 | 0 | 0 | 0 |
| 8d | 0 | 5 | 0 | 0 | 0 | 0 |
| 9d | 0 | 5 | 0 | 0 | 0 | 0 |
| 10d | 0 | 5 | 0 | 0 | 0 | 0 |
| 11d | 0 | 5 | 2 | 0 | 0 | 0 |
| 12d | 0 | 7 | 5 | 0 | 0 | 0 |
| 13d | 0 | 7 | 6 | 4 | 0 | 0 |
| 14d | 0 | 7 | 7 | 8 | 0 | 0 |
| 15d | 0 | 8 | 8 | 9 | 1 | 0 |
| 16d | 0 | 8 | 8 | 9 | 1 | 0 |
| 17d | 0 | 8 | 10 | 9 | 1 | 0 |
| 18d | 1 | 8 | 10 | 9 | 1 | 0 |
| 19d | 1 | 8 | 10 | 9 | 1 | 0 |
| 20d | 1 | 8 | 10 | 9 | 1 | 0 |

Supplemental figure 1. Determination of median lethal dose (LD₅₀) of *Nocardia seriolae* in *Lateolabrax maculatus*. Fish (n = 10 per dose group) were intraperitoneally injected with graded doses of *N. seriolae*: PBS (negative control), 5 × 10², 5 × 10³, 5 × 10⁴, 5 × 10⁵, and 5 × 10⁶ CFU per fish. Cumulative mortality was recorded over 20 days post-injection. The LD₅₀ was calculated by linear interpolation from the cumulative mortality data, yielding a value of 4.8 × 10⁴ CFU per fish.

|  | Water temperature (°C) |
| --- | --- |
| 1d | 28.1 |
| 2d | 28.9 |
| 3d | 28.6 |
| 4d | 28.5 |
| 5d | 27.8 |
| 6d | 28.1 |
| 7d | 27 |
| 8d | 26.9 |
| 9d | 28 |
| 10d | 28.7 |
| 11d | 28.8 |
| 12d | 28.9 |
| 13d | 28.9 |
| 14d | 29.2 |
| 15d | 27.5 |
| 16d | 28.1 |
| 17d | 28 |
| 18d | 27.7 |
| 19d | 27.2 |
| 20d | 27.1 |

Supplemental figure 2. Water temperature profile of the experimental system throughout the 20-day challenge period.
